# The Structural Dynamics of the Flavivirus Fusion Peptide–Membrane Interaction

**DOI:** 10.1371/journal.pone.0047596

**Published:** 2012-10-19

**Authors:** Ygara S. Mendes, Nathalia S. Alves, Theo L. F. Souza, Ivanildo P. Sousa, M. Lucia Bianconi, Rafael C. Bernardi, Pedro G. Pascutti, Jerson L. Silva, Andre M. O. Gomes, Andréa C. Oliveira

**Affiliations:** 1 Programa de Biologia Estrutural, Instituto de Bioquímica Médica, and Instituto Nacional de Ciência e Tecnologia de Biologia Estrutural e Bioimagem, Universidade Federal do Rio de Janeiro, Rio de Janeiro, Rio de Janeiro, Brazil; 2 Faculdade de Farmácia, Universidade Federal do Rio de Janeiro, Rio de Janeiro, Rio de Janeiro, Brazil; 3 Programa de Vacinas Virais, Instituto de Tecnologia em Imunobiológicos, Fundação Oswaldo Cruz, Rio de Janeiro, Rio de Janeiro, Brazil; 4 Instituto de Biofísica Carlos Chagas Filho, Universidade Federal do Rio de Janeiro, Rio de Janeiro, Rio de Janeiro, Brazil; Nagoya University, Japan

## Abstract

Membrane fusion is a crucial step in flavivirus infections and a potential target for antiviral strategies. Lipids and proteins play cooperative roles in the fusion process, which is triggered by the acidic pH inside the endosome. This acidic environment induces many changes in glycoprotein conformation and allows the action of a highly conserved hydrophobic sequence, the fusion peptide (FP). Despite the large volume of information available on the virus-triggered fusion process, little is known regarding the mechanisms behind flavivirus–cell membrane fusion. Here, we evaluated the contribution of a natural single amino acid difference on two flavivirus FPs, FLA_G_ (^98^DRGWGNGCGLFGK^110^) and FLA_H_ (^98^DRGWGNHCGLFGK^110^), and investigated the role of the charge of the target membrane on the fusion process. We used an *in silico* approach to simulate the interaction of the FPs with a lipid bilayer in a complementary way and used spectroscopic approaches to collect conformation information. We found that both peptides interact with neutral and anionic micelles, and molecular dynamics (MD) simulations showed the interaction of the FPs with the lipid bilayer. The participation of the indole ring of Trp appeared to be important for the anchoring of both peptides in the membrane model, as indicated by MD simulations and spectroscopic analyses. Mild differences between FLA_G_ and FLA_H_ were observed according to the pH and the charge of the target membrane model. The MD simulations of the membrane showed that both peptides adopted a bend structure, and an interaction between the aromatic residues was strongly suggested, which was also observed by circular dichroism in the presence of micelles. As the FPs of viral fusion proteins play a key role in the mechanism of viral fusion, understanding the interactions between peptides and membranes is crucial for medical science and biology and may contribute to the design of new antiviral drugs.

## Introduction

Enveloped viruses usually enter host cells through membrane fusion mediated by a viral glycoprotein that protrudes through the viral envelope [1]. All virus fusion-mediating glycoproteins share an internal sequence known as the fusion peptide (FP) that is highly conserved within the virus family [2]. The FP is responsible for the direct interaction of the fusion protein with the target membrane when the fusogenic conformation is achieved.

The peptide–membrane interaction can be divided into three thermodynamic steps: electrostatic attraction; transition into the binding surface; and a change in the peptide conformation. These steps lead to modifications in the thermodynamic parameters of the interaction. For this interaction to occur, the peptide charge, the hydrophobic/hydrophilic balance of the molecular groups, and the forces involved in the interaction are all relevant [3]. In addition, *in vitro* studies on membrane-destabilizing effects support the idea that lipids modulate the fusion of membranes with many viruses including influenza virus, Semliki Forest virus, Sendai virus, Ebola virus, and human immunodeficiency virus [4]–[12].

Flaviviruses are enveloped viruses belonging to the *Flaviviridae* family that are usually transmitted by hematophagous arthropods and are associated with high morbidity and mortality worldwide [13]. A transmembrane E glycoprotein mediates the fusion between the virus and the membrane of the target cell. This mechanism has been better described for the tick-borne encephalitis virus (TBEV) E glycoprotein, which is the prototype of this genus. In the mature virus, fusion proteins are present in the envelope as E-E homodimers that dissociate and form highly stable homotrimers after exposure to endocytic low pH, which is needed for fusion [14]. This conformational change exposes the FP, allowing the protein to interact with the membrane and destabilize it. The FP insertion in the lipid bilayer induces deformations in the target membrane, favoring fusion with the viral membrane. Thus, FP exposure is a key regulatory element of the fusion reaction. Nevertheless, the mechanisms used by FPs to accomplish this task remain poorly understood.

Crystallographic studies have revealed that the TBEV E protein ectodomain contains three domains, and the internal fusion peptide is in a CD loop at the tip of domain II, distantly located from the N-terminus [15]. It has been proposed that a highly conserved loop of the *Flavivirus* E protein, comprising amino acids 98–110, is the internal FP [16]. In mosquito-borne flaviviruses such as dengue (DV), yellow fever (YFV), and West Nile (WNV) viruses, the residue at position 104 is a glycine, whereas in tick-borne strains such as TBEV, it is a histidine [17].

Although much information has been gathered in recent years regarding the different conformations of *Flavivirus* fusion proteins, little is known regarding the mechanisms behind the virus–cell membrane fusion. Thus, elucidating the nature of the interactions between membrane fusion proteins and target membranes and the mechanisms by which these proteins promote the formation of membrane fusion intermediates is crucial for a better understanding of the virus fusion process.

Here, we investigated whether a natural single amino acid difference in the highly conserved flavivirus FP and the charges of the target membrane lipids affect the flavivirus fusion process. We studied the interaction between biomimetic membrane systems of different charges and different internal peptide sequences of the E structural proteins of flaviviruses. We used an *in silico* approach to simulate the interaction of FPs with a lipid bilayer in a complementary way with spectroscopic approaches to collect molecular parameters and conformational information. These data provide us new insights into the flavivirus fusion peptides–membrane interaction.

## Results

### Hydrophobicity Analysis of Fusion Peptides

Although the main mechanism of membrane fusion and the importance of the fusion peptide in this process have been accepted, the mechanism by which fusion peptides of *Flavivirus* execute this role remains elusive. Thus, to investigate the fusion peptide–target membrane interaction, we used two different internal peptide sequences of the E structural proteins of flaviviruses and biomimetic membrane systems.

**Figure 1:**
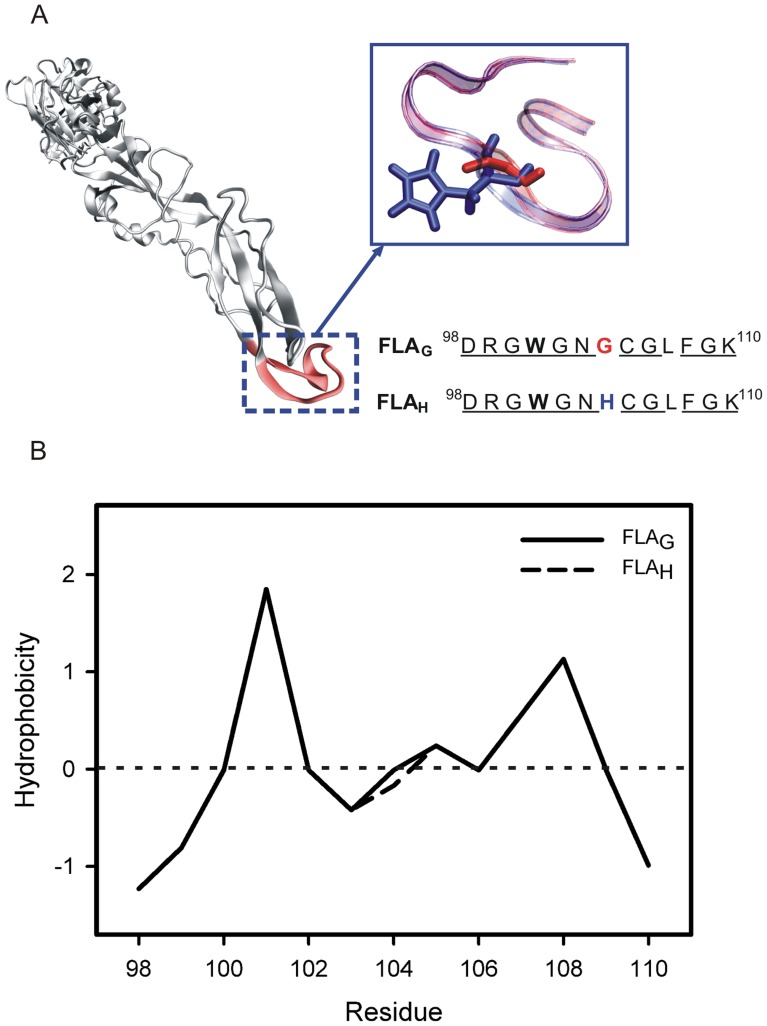
The flavivirus E glycoprotein fusion loop and hydrophobicity plots of the fusion peptides. (A) The crystallographic structure of West Nile virus E protein (PDB ID 2HG0) and the schematic representation and sequence of the two fusion peptides of flaviviruses studied in this work. FLA_G_ has a Gly residue (red) in position 104 of glycoprotein E, while FLA_H_ presents a His residue (blue). Trp101, Gly104 and His104 are indicated in bold, red and blue, respectively. The conserved amino acids are underlined. (B) Hydrophobicity plots for the fusion peptides FLA_G_ (solid line) and FLA_H_ (dashed line) were elaborated using the Wimley-White hydrophobicity scale.

The studied fusion peptides, FLA_G_ (^98^DRGWGNGCGLFGK^110^) and FLA_H_ (^98^DRGWGNHCGLFGK^110^), include two positively charged (Arg99 and Lys110), one negatively charged (Asp98) and two aromatic (Trp101 and Phe108) amino acids. At neutral pH, both peptides exhibit a positive charge of +1. When the pH is lower than 6, the peptide FLA_H_ acquires an additional charge (+2) due to the presence of a His residue at position 104 (Fig. 1A).

The tendency of the peptides to insert themselves into membranes was evaluated using the Wimley-White hydrophobicity plot [18] (Fig. 1B). The peptides presented with a slight hydrophobicity, except for the aromatic residues. A high tendency to partition into membranes is expected for the aromatic residues, but total insertion would not be favored due to the presence of charged residues and the hydrophilic profile of both ends of the peptides. Overall, the plot suggests a tendency for an interaction of the peptide with the membrane surface.

### Molecular Dynamics Simulations of Peptide–Membrane Interaction

**Figure 2:**
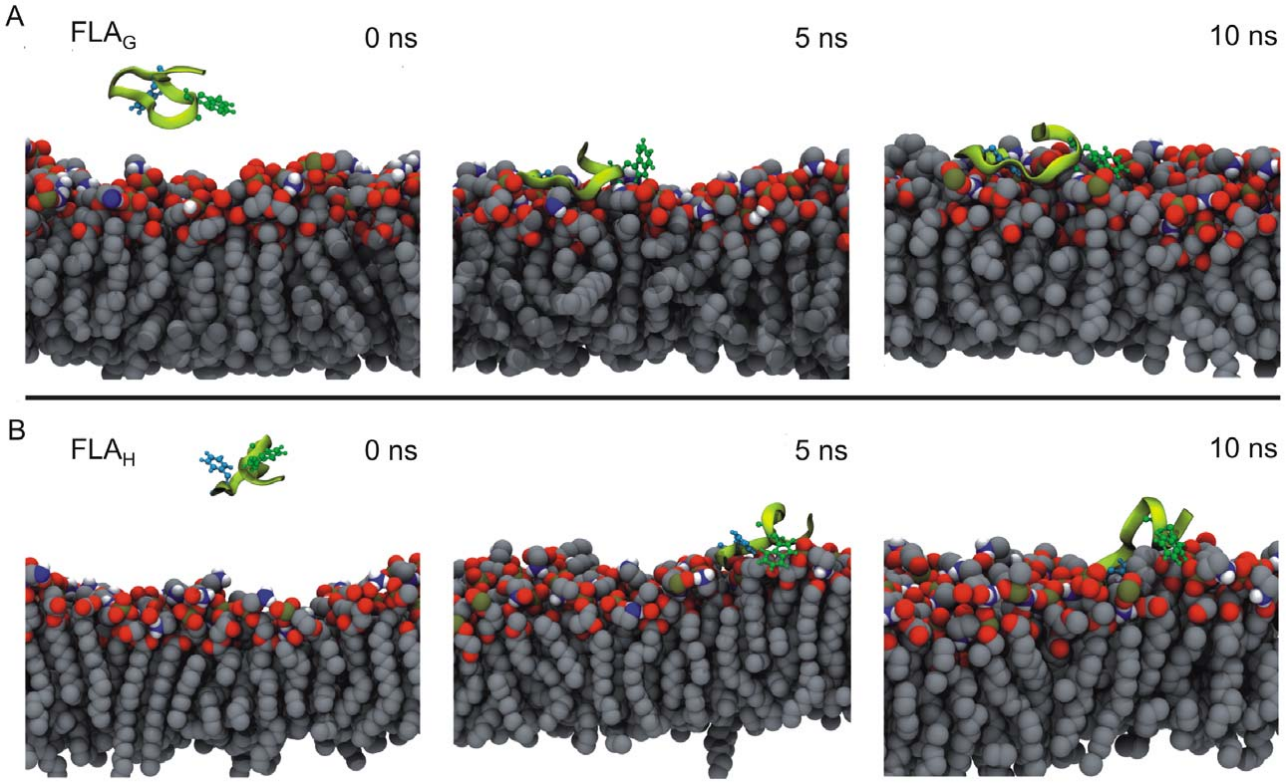
Molecular dynamics simulation of the interaction of the fusion peptides with the POPE membrane. Representative snapshots from simulations of FLA_G_ (A) and FLA_H_ (B) interaction with a POPE membrane at 35°C. The peptides are shown in light green, the membranes are in gray and, in each peptide, the side chains of residues Trp101 and Phe 108 are highlighted in dark green and blue, respectively.

**Figure 3:**
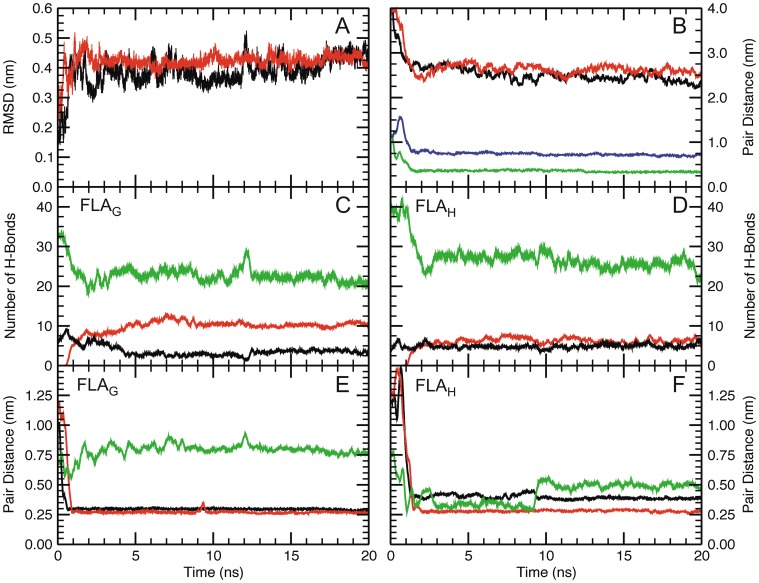
Molecular dynamics studies of the POPE membrane environment. (A) The RMSD of FLA_G_ (black) and FLA_H_ (red). (B) The distance between the peptide and membrane was determined as follows: the distance between the center of mass of the peptide and the membrane in the axis perpendicular to the membrane surface plane for both FLA_G_ (black) and FLA_H_ (red); and the minimal distance between Gly104 (green) or His104 (blue) atoms and the phosphorus atoms of the lipids. (C and D) The number of intramolecular hydrogen bonds (black) and those formed between the fusion peptides FLA_G_ (C) or FLA_H_ (D) and the water (green) or the POPE membrane (red). (E and F) The minimal distance between the Trp101 residue and the POPE membrane (red) and between the Phe108 residue and the POPE membrane (black) during MD simulation in the presence of the POPE membrane at 35°C. The intermolecular distance between Trp101 and Phe108 is also presented (green). The results for MD simulation of FLA_G_ and FLA_H_ are shown in E and F, respectively.

Seeking to understand the characteristics of the peptide–membrane interactions, molecular dynamics (MD) simulations of the peptides FLA_G_ and FLA_H_ were performed using a palmitoyl-oleoyl-phosphatidylethanolamine (POPE) lipid bilayer model (Fig. 2 and Fig. 3). Representative snapshots of the peptide–membrane interaction simulations are presented in Fig. 2. Plots showing the molecular parameters extracted from the simulations are shown in Fig. 3. During the simulation, the energy decreased as the peptides approached the membrane (data not shown), and the RMSD showed that the system reached equilibrium after 1 ns of simulation, suggesting that a simple hydrophobic model favors the interaction of both peptides with the membrane (Fig. 3A). The peptide–membrane interaction was defined as the distance from the nearest atom of each amino acid to the phosphorus atom of the lipids. The minimum distances between Gly or His (in FLA_G_ and FLA_H_ peptides, respectively) and the lipids’ polar head groups were reached after 1 ns, remaining as such until the end of the simulation, which agreed with the RMSD finding (Fig. 3B). The plot shows that the Gly residue at position 104 remained closer to the membrane than His. When the proximity of the whole peptide to the membrane was calculated by averaging the distances of all residues, we observed that the peptide FLA_G_ remained slightly closer to the membrane than the peptide FLA_H_.

The formation of hydrogen bonds during the simulation was also evaluated. Peptide–water and peptide intramolecular hydrogen bonds decreased early in the simulation process, while peptide–membrane hydrogen bonds increased. These changes were more evident for FLA_G_ than for FLA_H_ (Fig. 3C, D). Despite the observed changes, the peptide–water hydrogen bonds predominated during the entire simulation.

The analysis of the minimum distance between all amino acid residues of the peptide FLA_G_ and the membrane showed that the residues closer to the membrane were Trp101 and Phe108 (Fig. 3E). Both aromatic residues quickly reached the membrane–water interface and remained very stable during the residual simulation time. In the peptide FLA_H_, both aromatic residues quickly reached the membrane and remained attached. However, only Trp appeared to interact with the membrane as it approached the polar heads (Fig. 3F).

### Fluorescence Spectroscopy Analyses

The intrinsic fluorescence of the studied peptides was measured to evaluate the interaction with micelles of different detergents to confirm and complement the MD simulations data. Both peptides showed a maximum absorption at 280 nm and a maximum emission wavelength (λ_max_) at 349 nm when free in solution. While changes in the maximum emission intensity and λ_max_ position provide information regarding the main electronic transition observed for a fluorescent molecule, the analyses of the spectral center of mass and total spectral area are more representative of the global changes of the environment of the aromatic residues and the number of molecules contributing to the changes in fluorescence intensity [19]. Table 1 shows the shifts in spectral center of mass (ΔCM) and spectral area ratios (S/S_0_) for both peptides when in the presence of micelles.

**Table 1:** Fluorescence spectroscopy data of FLA_G_ and FLA_H_ in the presence of micelles.

	SDS		n-OGP	
	ΔCM (cm^−1^)	S/S_0_	ΔCM (cm^−1^)	S/S_0_
**FLA_H_ - pH 7.4**	772.3±40.9	1.7±0.5	104.1±50.1	1.3±0.1
**FLA_G_ - pH 7.4**	706.4±6.1	2.4±0.4	220.4±75.2	0.7±0.2
**FLA_H_ - pH 5.5**	742.6±22.9	1.8±0.2	79.2±15.3	1.2±0.4
**FLA_G_ - pH 5.5**	709.8±12.7	2.1±0.5	86.0±20.8	1.2±0.2
**λ_max_ blue shift (nm)** [1]	15		3	

All measurements were performed in triplicate in the same experiment, and the results were obtained from at least six independent experiments. ΔCM and S/S_0_ are expressed as the mean ± SD.

[1] Blue shift was determined by subtracting emission wavelength from control.

**Figure 4:**
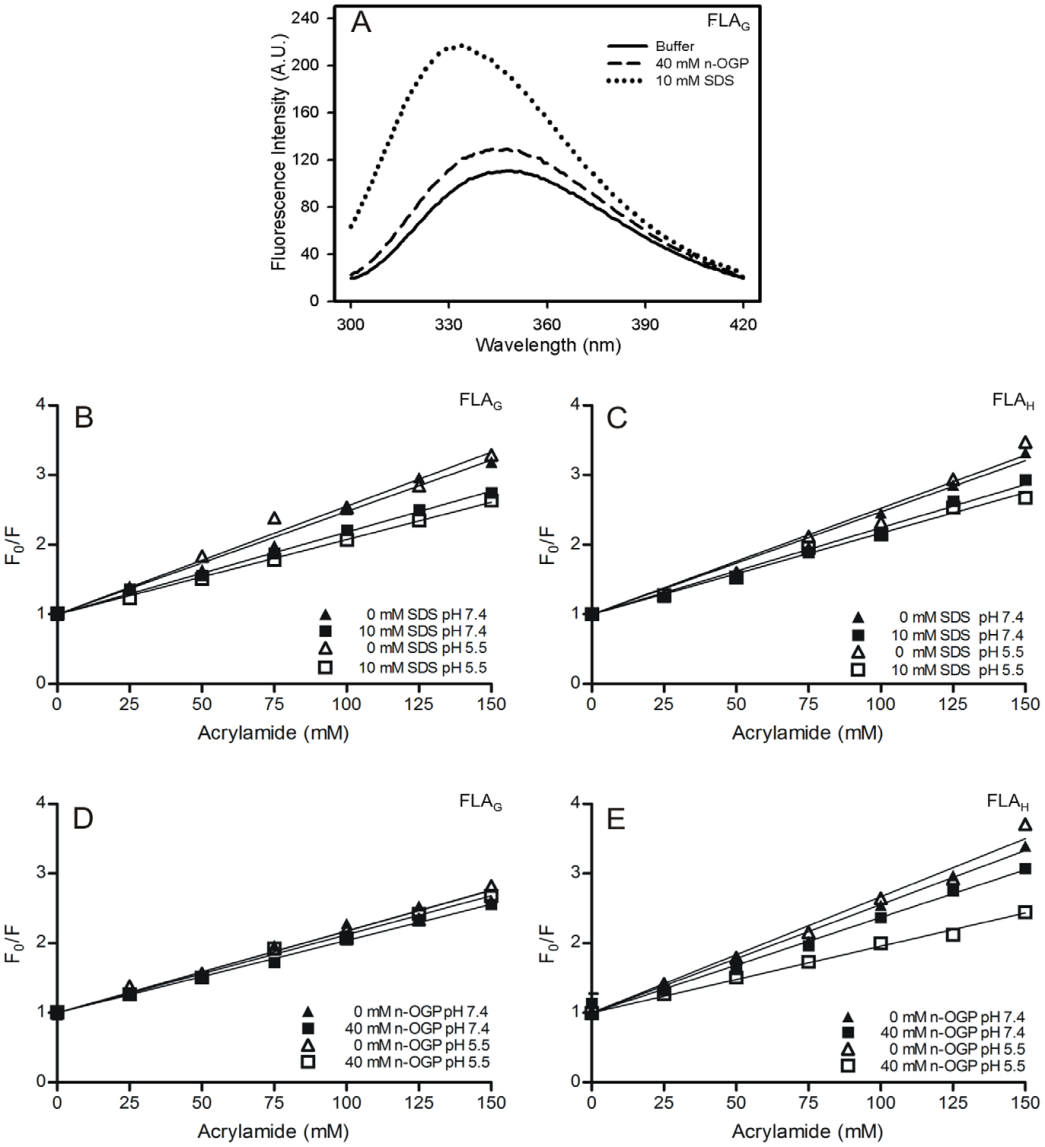
The fluorescence emission spectra of FLA_G_ and the analyses of FLA_G_ and FLA_H_ interactions with different micelles. (A) The fluorescence intensity emission spectra of FLA_G_ in the absence (solid line) or presence of SDS (dashed line) and n-OGP micelles (dotted line) at pH 7.4. (B–E) Stern-Volmer plots of fluorescence quenching of FLA_G_ and FLA_H_ by acrylamide at 37°C in micelle-free buffer (triangles) or in the presence of different micelles (squares). Stern-Volmer plots for FLA_G_ (B and D) and FLA_H_ (C and E) in the presence of 10 mM SDS (B and C) and 40 mM n-OGP (D and E). The pH 7.4 data are shown as closed symbols, and the pH 5.5 data as open symbols. FP fluorescence was recorded at 349 nm (micelle-free buffer), 334 nm (in the presence of SDS micelles), and 346 nm (in the presence of n-OGP micelles).

In the presence of n-OGP neutral micelles, the λ_max_ of the peptide FLA_G_ at pH 7.4 presented with a blue shift of 3 nm (349 to 346 nm) and a ΔCM of 220 cm**^−^** ^1^. No increase in fluorescence intensity was observed (Fig. 4A and Table 1). In contrast, the fluorescence emission of FLA_G_ in the presence of anionic micelles (SDS) showed that the λ_max_ shifted to approximately 334 nm, with a ΔCM of 772.3 cm**^−^** ^1^ (Fig. 4A and Table 1). Increases of approximately three times the intensity at λ_max_ and 2.4 times the spectral area were observed (Table 1). Similar results were obtained for the peptide FLA_H_, and there was no significant difference between pH 7.4 and 5.5 for either peptide. The greater prominent blue shift and increased intensity suggest a deeper penetration of the Trp residues into SDS micelles than n-OGP micelles. The variation of the spectral center of mass reflects more directly the changes in the environment around the aromatic residues of the peptides. The changes in intensity represented by the S/S_0_ ratio are related to changes in the environment and also reflect the increased peptide fraction bound to micelles. Although it is difficult to quantitatively separate both effects in our data, the more than two-fold increase in fluorescence intensity observed for both peptides in the presence of SDS suggests that there is a higher peptide fraction bound to the anionic micelles.

The depth of penetration of Trp residues in the hydrophobic core of SDS and n-OGP micelles was evaluated by a fluorescence-quenching experiment using acrylamide. Fluorescence quenching depends on different characteristics of the system, such as the diffusion constants of the quencher in water and in the micelle, the nature of the interaction of the fluorescent peptide with the micelles, the lateral interactions between detergent molecules and water penetration in the micelle hydrophobic core. The intrinsic fluorescence of both peptides obeyed the linear Stern-Volmer equation when acrylamide was used as a neutral hydrophilic and dynamic quencher (Fig. 4B, C, D, E). Peptide fluorescence quenching was measured at pH 7.4 and pH 5.5 in micelle-free buffer solution or in the presence of n-OGP (Fig. 4B, C) or SDS (Fig. 4D, E) micelles. The Stern-Volmer constant values of all conditions are shown in Table 2.

**Table 2:** Fusion peptides binding to different micelles as measured by acrylamide quenching.

	Calculated Ksv (M^−1^)				
Condition	Buffer	SDS (10 mM)	p value[^1^]	n-OGP (40 mM)	p value[^2^]
**FLA_G_ in pH 7.4**	12.9±0.3	11.4±0.3	0.005**	10.6±0.2	<0.0001***
**FLA_G_ in pH 5.5**	12.6±0.4	11.0±0.2	0.0209*	11.7±0.2	0.2087^ns^
**FLA_H_ in pH 7.4**	15.0±0.2	12.2±0.1	<0.0001***	14.3±0.2	0.0359*
**FLA_H_ in pH 5.5**	16.1±0.3	12.2±0.4	<0.0001***	11.3±0.7	<0.0001***

All measurements were performed in triplicate in the same experiment, and the results were obtained from at least six independent experiments. The Ksv values are expressed as the mean ± SEM. The significance coefficient was obtained using the paired Student’s t test. p values were calculated by buffer-SDS[^1^] and buffer-n-OGP[^2^] pairs. ns – not significant;

* 0.01<p<0.05 (significant);

** 0.001<p<0.005 (very significant);

*** p<0.001 (extremely significant).

In micelle-free buffer, the peptides presented a Stern-Volmer quenching constant of approximately 12 M**^−^** ^1^ and 16 M**^−^** ^1^ for FLA_G_ and FLA_H_, respectively (Table 2). In the presence of micelles, the extent of quenching decreased, suggesting a partial protection from the aqueous solvent (Fig. 4B, C, D, E and Table 2). The changes observed for the quenching constants of FLA_H_ and FLA_G_ in the presence of all micellar solutions tested were significant, except for the peptide FLA_G_ in the presence of n-OGP micelles at pH 5.5 (Table 2).

In addition to the possible differences in peptide behavior, the micelles may offer very different environments for peptide interactions. The main differences are related to the hydrophobic core and the presence or absence of surface charges. The micelles used here included the negatively charged SDS micelles and the neutral n-OGP micelles. To evaluate the differences in water penetration and lateral interactions in both micelles, we used Laurdan fluorescence analyses. Laurdan is a fluorescent lipophilic probe that detects changes in the polarity of its environment when it is bound to either membranes or micelles [20], [21]. Variations in the access of water molecules to the hydrophobic core of membranes and micelles cause shifts in the Laurdan fluorescence emission spectrum. Blue shifts can be directly related to the exclusion of water molecules due to strong lateral interaction among lipid or detergent molecules. The quantification of these changes can be achieved by computing the Generalized Polarization (GP) value, which can theoretically range from −1, corresponding to most fluid or polar environment, to +1, corresponding to most condensed or nonpolar environment [22].

When incubated in the presence of Laurdan, the SDS micelles showed Laurdan GP values of 0.34 and 0.21 at pH values of 7.4 and 5.5, respectively, while n-OGP micelles showed GP values of −0.53 and −0.61 at the same respective pH values (Laurdan spectra not shown). These results show that pH levels do not change the water penetration in the micelles and indicate that the n-OGP micelles allow for the penetration of water to a substantially higher extent than SDS micelles. Compared to membranes, the water content in the hydrophobic core of SDS micelles is similar to liquid-ordered membrane regions, while n-OGP micelles are more similar to fluid disordered membrane regions [23], [24]. This is in agreement with the observation of very small changes in the intrinsic fluorescence of the peptides in the presence of n-OGP micelles. Although the interaction of the neutral n-OGP micelles with the uncharged FLA_G_ peptide is not expected to be affected by changes in pH, the less packed and largely hydrated environment provided by n-OGP micelles may be responsible for the observation of non-significant protection of the peptide from acrylamide quenching at pH 5.5, as these conditions would cause a faster diffusion of acrylamide and peptides in and out of the micelles during equilibrium.

Fluorescence spectroscopy data confirmed the interaction of the peptides with biomimetic membrane models, as suggested by MD simulations, and indicated a stronger interaction of the peptides with SDS micelles.

### Conformational Changes of the Fusion Peptides Induced by Interaction with Membrane Models

**Figure 5:**
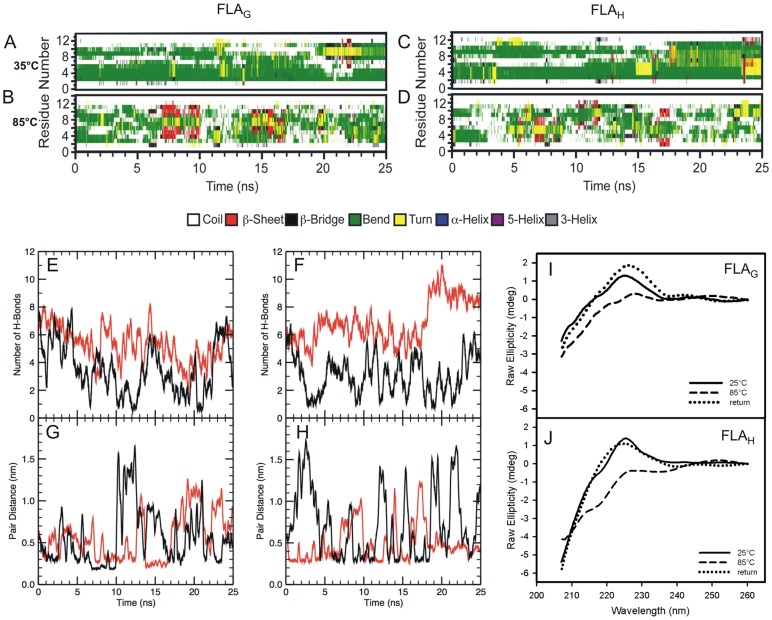
The secondary structure of peptides FLA_G_ and FLA_H_ in aqueous buffer. The secondary structure patterns of the fusion peptides FLA_G_ (A) and FLA_H_ (B), at 35°C and secondary structure patterns of the FP FLA_G_ (C) and FLA_H_ (D) at 85°C in solution. (E and F) The MD simulation of FLA_G_ (E) and FLA_H_ (F) in water at 35°C (red line) and at 85°C (black line). (G and H) The minimal distance between FLA_G_ (G) or FLA_H_ (H) Trp101 and Phe108 residues at 35°C (red line) and at 85°C (black line). (I and J) The circular dichroism spectra of the FLA_G_ (I) and FLA_H_ (J) fusion peptides in solution at 25°C (solid line), at 85°C (dashed line), and the return to 25°C (dotted line). The experiments were performed at room temperature at pH 5.5.

Viral protein structural changes are expected during the fusion process when either the change in pH or the interaction with the membrane leads to the final fusogenic conformation. MD simulations and circular dichroism (CD) measurements were used to evaluate a possible gain of structure during the interaction of FLA_G_ and FLA_H_ with membrane models. The MD simulations indicated that both peptides adopted a bent structure when in aqueous solution at 35°C (Fig. 5A, C). However, the N- and C-terminal residues were very unstable, presenting random structures throughout the simulation time. Moreover, the CD data revealed that the peptides FLA_G_ and FLA_H_ exhibited a random coil conformation in aqueous solution and showed an unusual positive peak at 225–230 nm (Fig. 5I, J), indicating intense exciton-coupled bands. This profile has been previously described for other peptides [25] and indicates an interaction between aromatic chromophores. Thus, our result suggests that Trp101 and Phe108 are likely to be interacting and that both peptides assume a bent conformation.

To investigate whether an interaction between the aromatics was occurring, both peptides were subjected to high temperatures to disrupt the possible weak interactions present in each structure. CD measurements for both peptides FLA_G_ and FLA_H_ at 85°C showed that the positive peak near 225 nm disappeared in a reversible manner (Fig. 5I, J). This fact corroborates our hypothesis that FLA_G_ and FLA_H_ both adopt a bent conformation.

As the CD data suggested a bent conformation, as evidenced by the interaction between the aromatic residues Trp101 and Phe108, simulations were also performed at 85°C (Fig. 5B, D). Evaluating the secondary structure under these conditions, we observed that both peptides remained very unstable with many momentary fluctuations of secondary structures and prevalent random conformations.

Non-covalent interactions such as hydrogen bonds are essential for maintaining protein structure. The interaction stability present in these two systems could be better viewed by the formation/breaking of hydrogen bonds throughout the simulation (Fig. 5E, F). The disruption of hydrogen bonds at 85°C was more evident for the peptide FLA_H_. The interaction between the aromatic residues was determined by a minimum distance value between atoms, which is indicative of interaction (Fig. 5G, H). Comparing the simulations performed at 35°C and 85°C, we observed the stabilization of the structure at 35°C because the high temperature is capable of breaking the hydrogen bonds, which disfavors the approximation between the residues Trp101 and Phe108.

**Figure 6:**
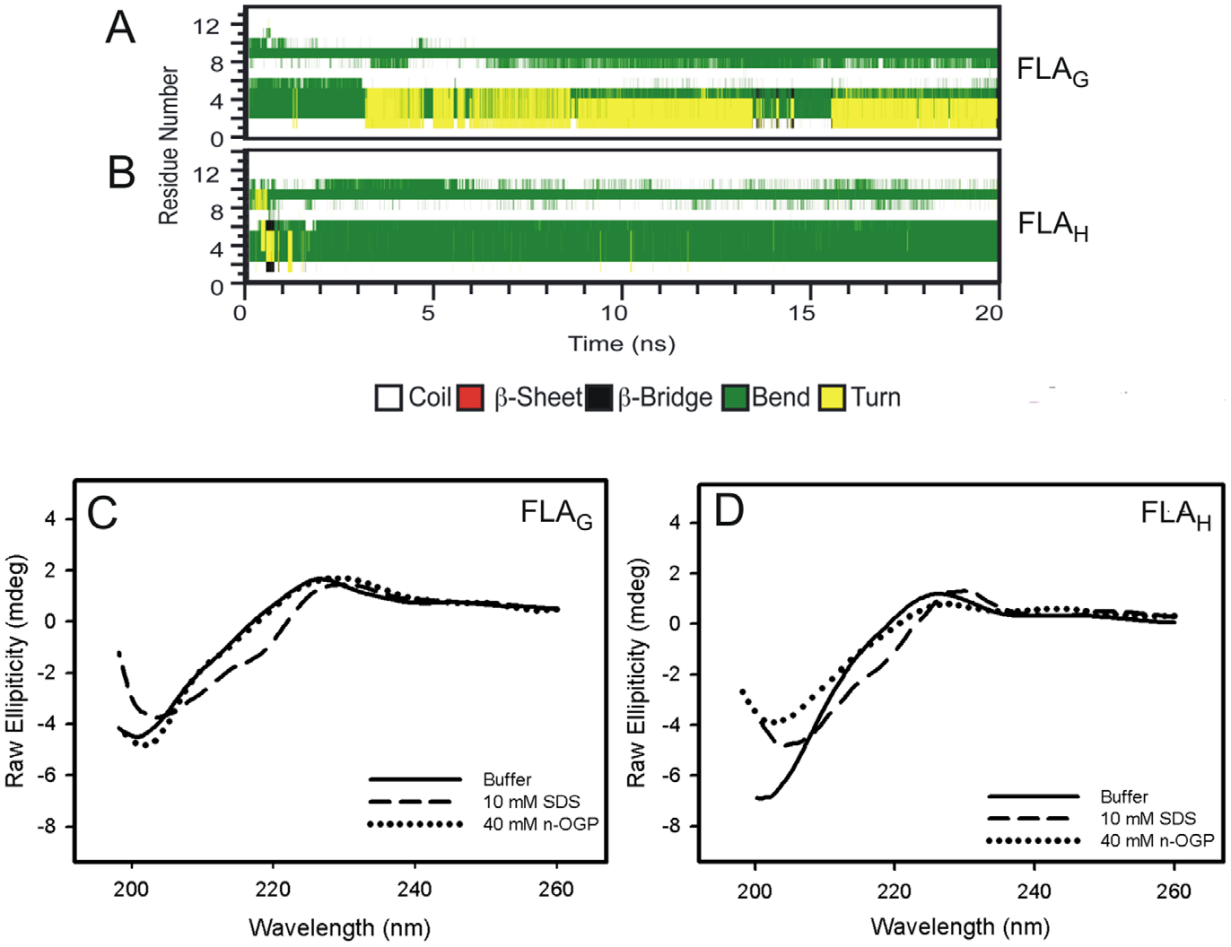
The secondary structures of FLA_G_ and FLA_H_ in the presence of membrane models. (A and B) The secondary structures of the fusion peptides FLA_G_ (A) and FLA_H_ (B) in the presence of POPE membranes at 35°C. (C and D) The circular dichroism spectra of the FLA_G_ (C) and FLA_H_ (D) FP in solution (solid line) and in the presence of SDS (dashed line) or n-OGP (dotted line) micelles. The experiments were performed at room temperature at pH 5.5.

MD simulations of peptides FLA_G_ and FLA_H_ in the presence of a lipid bilayer showed that the bend structure adopted by the peptides remained fairly stable, indicating that the membrane environment stabilized the fusion peptide conformation (Fig. 6A, B). The CD spectra of both peptides in the presence of SDS micelles showed a positive shift in the region near 218 nm (Fig. 6C, D), indicating an increase in the secondary structure. These changes were not observed in the presence of n-OGP micelles (Fig. 6C, D).

## Discussion

Here, we evaluated the contribution of a single amino acid difference on two flavivirus fusion peptides and the importance of the target membrane charge to the fusion process. We found that both peptides interacted with different membrane models at the water–membrane interface and adopted a bent structure. Mild differences between FLA_G_ and FLA_H_ were observed depending on the pH and the charge of the target membrane.

According to the current model of viral fusion, fusion peptides bear an intrinsic capacity to disrupt the target bilayer architecture after insertion and to directly mediate membrane merging [2], [9], [26]. Usually, insertion of peptides into the lipid bilayers of cellular target membranes and the gain of structure are concerted processes.

Our results indicate that the peptides FLA_G_ and FLA_H_ interact with neutral and anionic micelles in different ways. The greater energy conservation resulting from the peptide–SDS micelle interaction is revealed by a higher increase in the peptide fluorescence intensity and by a greater blue shift of its fluorescence spectra. This fact may be explained by a difference in the environment of the peptide tryptophan residue that occurs due to the peptide–SDS micelle interaction. These results suggest that SDS micelles favor interaction with peptides, as the depth of penetration of the Trp residues into the hydrophobic core of the micelles appears to be higher in the SDS micelles than in the n-OGP micelles. This indicates that peptide–membrane model interactions can be influenced by micelle charge. The higher changes in intensity represented by S/S_0_ ratios are related to changes in the environment and may also be the result of a larger fraction of peptides bound to the micelle. The more than two-fold increase in fluorescence intensity observed for both peptides in the presence of SDS suggests that there is a larger peptide fraction bound to the anionic micelles. As shown by the Laurdan GP data, the environments in the micelles are rather different, making it difficult to quantitatively separate both effects in our data to point to a difference in partition coefficients. However, we speculate that the binding to SDS micelles is more stable than the binding to n-OGP micelles. The high hydration and loose packing of the n-OGP micelles may explain why the FLA_G_ with n-OGP micelles at pH 5.5 is the only condition that showed no significant protection from acrylamide quenching, as the diffusion of acrylamide and the peptides appears to be faster between the aqueous solvent and the micelle under this condition. The protection from quenching provided by SDS micelles indicates a smaller diffusion of peptides and quencher at the micelle/water interface. These data reinforce the more favorable interaction of the peptides with SDS than with n-OGP micelles.

The presence of His104 in the peptide FLA_H_ does not appear to influence the interaction with neutral membranes, as no differences were observed for the interaction of FLA_H_ and FLA_G_ with neutral micelles in acidic or neutral pH. It has recently been proposed that the flavivirus histidines function as molecular switches, and they therefore control the fusion process by acting as pH sensors in the *Flavivirus* membrane fusion process [27]. However, a recent work publication showed that His104 protonation does not inhibit the ability of TBEV FP to bind to membranes [28]. Indeed, the data presented here, as well as in other studies, suggest that this is not the case for His104 in TBEV FP. In DENV, position 104 is a Gly, and mutations at this position indicate that the absence of a side chain is critical to DENV infection of vertebrate cells [29]. Our MD simulation data showed that Gly of FLA_G_ remained closer to the lipid bilayer than did His in FLA_H_, and when the average of the distances of all residues was calculated, FLA_G_ remained slightly closer to the membrane than FLA_H_.

During MD simulations, both peptides quickly reached the membrane and remained at the water–membrane interface as demonstrated by the predominance of peptide–water hydrogen bonds. Although changes in Stern-Volmer constants were significant, confirming the interaction between peptides and micelles, the values were only slightly reduced. Together, our data strongly suggest that both peptides interact on the surface of both micelles.

Both the burying of the indole ring of the tryptophan residue on the hydrophobic core and the electrostatic interaction on the micelle surface appear to be important for the stabilization of the interaction. Recently, it was shown that a flavivirus fusion peptide 15 residues long could only promote the fusion of vesicles containing the anionic phospholipid phosphatidylglycerol [30]. We speculate that if the interaction with neutral micelles misses the electrostatic component, the peptide–neutral micelle interaction is less efficient in inducing membrane fusion.

We also observed that Trp101 and Phe108 were closer to the membrane compared to other residues, which is consistent with hydrophobicity characteristics of peptides and reinforces previous observations that the hydrophobic patch formed by Trp101 and Phe108 is a putative structural motif responsible for membrane fusion in the Dengue dengue virus [30], [31]. The Trp-Phe interaction is also crucial for the interaction between the Ebola virus fusion domain and lipid rafts [11], [12]. In addition, Trp residues have been found to play crucial roles in the activity of several antimicrobial peptides, promoting the insertion of the peptide into the membrane interface [32]. Although the peptide–membrane interaction appears to be facilitated by the aromatic residues, this interaction may occur with or without Trp participation.

Peptides FLA_G_ and FLA_H_ predominantly exhibited a random coil structure when free in solution. This conformational flexibility can be explained by the high content of glycines present in the peptide structure and appears to be critical for the membrane fusion process of many viruses [33]. However, it has been shown that a fusion peptide 12 residues long weakly promotes membrane fusion, likely because of this flexibility. Electrostatic components are thus important to stabilize a conformation allowing a deeper insertion of the peptide [34].

An interaction between the aromatic residues was also strongly suggested by the observation of exciton-coupled bands in the CD spectra. The existence of this interaction was reinforced by the bend conformation observed in MD simulations, as aromatic residues Trp101 and Phe108 are distant in amino acid sequence. Because these non-covalent interactions can be disrupted at high temperature [35], the reversible loss of the characteristic signal of exciton-coupled band in the CD spectrum at 85°C confirmed the interaction between the aromatic residues. The MD simulations showed that high temperature promoted the disruption of intramolecular interactions such as hydrogen bonds, thereby separating the Trp and Phe residues, leading to an unstable structure with a more relaxed conformation. The effect of aromatic–aromatic interaction also appears to be important for maintaining the stability of secondary structure in the Ebola virus fusion peptide [11], [12].

In the presence of membranes, the bent structures of FLA_G_ and FLA_H_ remained stable during the simulation, indicating that the membrane environment stabilizes the fusion peptide conformations. Our results indicate that both peptides adopted a bent structure when in the presence of target membranes regardless of having a Gly or His at position 104. Note that an increase in secondary structure content was observed in the presence of SDS micelles but not in the presence of n-OGP micelles, indicating that the charge of the micelle appears to be important to stabilize the peptide–micelle interaction and is consequently important for the FP secondary structure gain. This increase is characteristic of a β structure, although we cannot confirm that any of the peptides actually adopt a β-hairpin structure. Nevertheless, these data corroborate the idea that the peptide–micelle interaction occurs in different ways when different micelles are present.

In conclusion, the conformation of these peptides when bound to mimetic membranes supports the contribution of electrostatic interactions with the negatively charged lipid head groups and of hydrophobic interactions with the tails of membrane fatty acids. Taken together, our results suggest that fusion of flaviviruses is promoted by the binding of fusion peptides to the surface of the lipid bilayer and their concerted gain of structure. Furthermore, the presence of indole rings of the Trp residues on the surface of the target membrane drives the peptide–membrane association process.

This study gives new insights into the mode of action of flavivirus fusion peptides. As the fusion peptides of viral fusion proteins play a key role in the mechanism of viral fusion, understanding the interactions between peptides and membranes is crucial for medical science and biology, contributing to the design of new antiviral drugs.

## Materials and Methods

### Materials and Reagents

Sodium dodecyl sulfate (SDS) and n-octyl-D-β-glucopyranoside (n-OGP) were obtained from Sigma Aldrich (St. Louis, MO, USA). Acrylamide and 6-dodecanoyl-2-dimethylaminonaphthalene (Laurdan) were purchased from Amersham Biosciences (Piscataway, NJ, USA) and Invitrogen (Carlsbad, CA, USA), respectively. Water was deionized and purified in Milli-Q equipment (Millipore, Molsheim, France). All reagents were of analytical grade.

### Peptides

The peptides ^98^DRGWGNGCGLFGK^110^ (FLA_G_) and ^98^DRGWGNHCGLFGK^110^ (FLA_H_) were synthesized by Genemed Synthesis Inc. (South San Francisco, CA, USA). The identity and purity (>95%) were established by amino acid sequencing, mass spectrometry, and high performance liquid chromatography analysis. Peptide stock solutions were prepared by suspending peptides in 20 mM sodium phosphate buffer, pH 7.4, in which they are fully soluble, and were stored at −20°C for up to one week. The peptide absorbance in aqueous solution was determined at 280 nm using a molar extinction coefficient (ε) of 5500 M**^−^** ^1^ cm**^−^** ^1^ to establish the peptide concentration.

### Fluorescence Spectroscopy

Fluorescence analyses were performed on an ISS K2 spectrofluorometer (ISS Inc., Champaign, IL, USA) at 37°C. An excitation wavelength of 280 nm was used to detect emissions between 300 to 420 nm in 1-nm increments. Excitation and emission slits of 2 and 1 nm, respectively, were used for measuring peptides in the presence and absence of detergent micelles. The spectral area data were obtained from the S/S_0_ ratio, where S_0_ and S represent the spectral area in the absence and presence of each detergent. The changes in the fluorescence spectra were evaluated by the changes in spectral center of mass, <ν>:
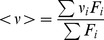
(1)where F*_i_* stands for the fluorescence emitted at wavenumber ν*_i_*, and the summation is performed over the range of appreciable values of F. The changes in the spectral center of mass were calculated by subtracting the values obtained in the absence of detergents from the values in the presence of detergents and were represented as ΔCM.

The peptides were diluted in 20 mM sodium phosphate buffer, pH 7.4 and 5.5, to a final concentration of 10 µM. The micelle solution stock was prepared as 100 mM SDS diluted in the same buffer at each of the two pH values, whereas n-OGP was prepared in water and varied approximately 1.5 M to ensure that concentrations of stock solutions were above the critical micelle concentration (CMC). The pH values were measured before and after each experiment.

### Acrylamide Quenching Measurements

For the tryptophan fluorescence quenching experiments, increasing concentrations of acrylamide were added from a 5 M stock solution to peptide solutions with or without micelles. We used an excitation wavelength of 280 nm, and fluorescence intensities at 349 nm (micelle-free buffer), 334 nm (for SDS micelles), and 346 nm (for n-OGP micelles) were monitored after each acrylamide addition at 37°C. A quenching constant Ksv was obtained using the Stern-Volmer equation for dynamic processes [19], [36].

Statistical analyses were performed using Student’s t test for comparing means between micelle-free samples and in the presence of SDS or n-OGP micelles. The software was Graph Pad Prism 4.0 (San Diego, CA, USA). For all comparisons, a p value less than 0.05 was regarded as significant.

### Circular Dichroism Spectroscopy

Circular dichroism (CD) measurements were performed with a Jasco J-715/1505 CD spectropolarimeter (Hachioji, Tokyo, Japan) between 190 to 260 nm, considering a 0.2-nm step resolution at 50 nm/min speed and using a cylindrical quartz with a 0.02-cm path length cuvette. The response time was 8 s, with 100 mdeg sensitivity and a 2 nm bandwidth. Each spectrum is the average of 10 independent scans. The peptide concentration was 2 mM, and the CD spectra were obtained at room temperature (25°C) in 20 mM sodium phosphate buffer, pH 5.5, with and without micelles. The contributions from background signals were subtracted from the CD spectra acquired for the peptides.

### Molecular Dynamics Simulations

Molecular dynamics (MD) simulations were performed for FLA_G_ and FLA_H_ both in a water box and in a fully solvated phospholipid membrane using the GROMACS package [37] in an NPT ensemble. The simulation time was 25 ns. To obtain the coordinates of the fusion peptide FLA_G_, we used the structure with the lowest energy, acquired from the crystallographic structure of WNV E protein (PDB ID 2HG0). The same protein coordinates used for FLA_G_ were used for FLA_H_, but the Gly residue at position 104 was replaced by a His residue, using a steepest descend method to obtain the structure around the mutated residue, minimizing the energy.

A fully hydrated 340 palmitoyl-oleoyl-phosphatidylethanolamine (POPE) membrane was obtained from Tieleman’s work [38]–[40]. The simple point charge (SPC) water model [41] was adopted in all simulations together with the GROMOS45A3 force field [42]. Counter ions were used to maintain the neutrality of the system charge, and Na^+^ and Cl^-^ ions were used to simulate a 150 mM NaCl solution.

The system was thermodynamically coupled at 35°C and at 85°C using a Berendsen thermostat [43] applied at each 0.1 ps. A Parinello-Rahman barostat [44] was used to couple pressure at 1.0 bar isotropically in the water box and semi-isotropically in the membrane system. The Lennard-Jones interactions were simulated by the switch function at a radius of 1 nm. The electrostatic interactions were considered up to 1.1 nm using the Particle-Mesh Ewald method [45] because, with the exception of His104 of the fusion peptide FLA_H_, which was protonated to simulate an acidic environment (pH <6), the residues were ionized at pH 7. The minimum distance analyses were based on the smallest distance between atoms of each peptides or membrane.

### Laurdan GP Function

The packing level of the micelles was inferred by means of Laurdan fluorescence. The samples were incubated with 20 µM Laurdan, excited at 350 nm, and the fluorescence emission was scanned from 380 to 600 nm. The excitation and emission slits were set in the range of 0.5 and 1.0, respectively. The assay was performed at 37°C. All data were recorded with adequate correction, averaged and normalized. Generalized polarization (GP) values were calculated as a function of the fluorescence emission intensities at 440 and 490 nm, based on Eq 2 and Eq 3:
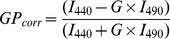
(2)where



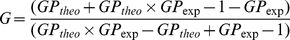
(3)I_440_ and I_490_ are the emission intensities at 440 and 490 nm, respectively, GP_theo_ corresponds to the known GP value of a standard solution of Laurdan in DMSO at 22°C (0.207), and GP_exp_ corresponds to the experimentally determined GP value of the standard solution [22].
